# Ischemia of the lung causes extensive long-term pulmonary injury: an experimental study

**DOI:** 10.1186/1465-9921-9-28

**Published:** 2008-03-26

**Authors:** Niels P van der Kaaij, Jolanda Kluin, Jack J Haitsma, Michael A den Bakker, Bart N Lambrecht, Burkhard Lachmann, Ron WF de Bruin, Ad JJC Bogers

**Affiliations:** 1 Department of Cardio-Thoracic Surgery, Erasmus MC, Rotterdam, the Netherlands; 2 Department of Cardio-Thoracic Surgery, Erasmus MC, Rotterdam, the Netherlands; at present at work at the department of Cardio-Thoracic Surgery, UMC Utrecht, Utrecht, the Netherlands; 3 Department of Anesthesiology, Erasmus MC, Rotterdam, the Netherlands; at present at work at the interdepartmental division of Critical Care, University of Toronto, Toronto, Canada; 4 Department of Pathology, Erasmus MC, Rotterdam, the Netherlands; 5 Department of Pulmonary Medicine, Erasmus MC, Rotterdam, the Netherlands; at present at work at the department of pulmonary medicine, University Hospital Gent, Gent, Belgium; 6 Department of Anaesthesiology, Erasmus MC, Rotterdam, the Netherlands; 7 Department of Surgery, Erasmus MC, Rotterdam, the Netherlands; 8 Department of Cardio-Thoracic Surgery, Erasmus MC, Rotterdam, the Netherlands

## Abstract

**Background:**

Lung ischemia-reperfusion injury (LIRI) is suggested to be a major risk factor for development of primary acute graft failure (PAGF) following lung transplantation, although other factors have been found to interplay with LIRI. The question whether LIRI exclusively results in PAGF seems difficult to answer, which is partly due to the lack of a long-term experimental LIRI model, in which PAGF changes can be studied. In addition, the long-term effects of LIRI are unclear and a detailed description of the immunological changes over time after LIRI is missing. Therefore our purpose was to establish a long-term experimental model of LIRI, and to study the impact of LIRI on the development of PAGF, using a broad spectrum of LIRI parameters including leukocyte kinetics.

**Methods:**

Male Sprague-Dawley rats (n = 135) were subjected to 120 minutes of left lung warm ischemia or were sham-operated. A third group served as healthy controls. Animals were sacrificed 1, 3, 7, 30 or 90 days after surgery. Blood gas values, lung compliance, surfactant conversion, capillary permeability, and the presence of MMP-2 and MMP-9 in broncho-alveolar-lavage fluid (BALf) were determined. Infiltration of granulocytes, macrophages and lymphocyte subsets (CD45RA^+^, CD5^+^CD4^+^, CD5^+^CD8^+^) was measured by flowcytometry in BALf, lung parenchyma, thoracic lymph nodes and spleen. Histological analysis was performed on HE sections.

**Results:**

LIRI resulted in hypoxemia, impaired left lung compliance, increased capillary permeability, surfactant conversion, and an increase in MMP-2 and MMP-9. In the BALf, most granulocytes were found on day 1 and CD5^+^CD4^+ ^and CD5^+^CD8^+^-cells were elevated on day 3. Increased numbers of macrophages were found on days 1, 3, 7 and 90. Histology on day 1 showed diffuse alveolar damage, resulting in fibroproliferative changes up to 90 days after LIRI.

**Conclusion:**

The short-, and long-term changes after LIRI in this model are similar to the changes found in both PAGF and ARDS after clinical lung transplantation. LIRI seems an independent risk factor for the development of PAGF and resulted in progressive deterioration of lung function and architecture, leading to extensive immunopathological and functional abnormalities up to 3 months after reperfusion.

## Background

Lung transplantation is currently an accepted treatment option for patients with end-stage pulmonary diseases, even though the outcome remains limited [1]. Development of primary acute graft failure (PAGF) occurs in 15–30% of lung transplant recipients and is the main cause for early morbidity and mortality after lung transplantation, resulting in a one-year survival rate of approximately 80% [1-3]. Lung ischemia reperfusion injury (LIRI) has been suggested to be a major risk factor for PAGF, although other factors like donor brain death, mechanical ventilation, pneumonia, hypotension, aspiration, donor trauma and allo-immunity have been found to interplay with LIRI in PAGF development [1-4]. The clinical expression of LIRI may range from mild hypoxemia and mild pulmonary edema on chest X-ray to PAGF, which is the most severe form of injury [1]. Symptoms of PAGF usually develop within 72 hours after reperfusion and consist of hypoxemia, which cannot be corrected by supplemental oxygen, non-cardiogenic pulmonary edema, increased pulmonary artery pressure, and decreased lung compliance [1,3-5].

Even though a positive correlation between cold ischemia time and PAGF development has been suggested [3,6-8], other studies found that duration of cold ischemia did not predict outcome after lung transplantation and suggested that other factors interplay with LIRI in PAGF development [9-14]. The question whether LIRI is an independent risk factor for the development of PAGF seems difficult to answer. In clinical studies, often multiple interfering factors are examined simultaneously. Furthermore, a long-term experimental LIRI model, in which PAGF changes can be studied, is missing. The majority of experimental studies use ex vivo LIRI models, like the Langendorff system, which is a non-physiological model and in which it is impossible to investigate reperfusion times beyond the first hours. In addition, an experimental lung transplantation model with the induction of cold ischemia is technically difficult in rodents. Thus, the purpose of this study was to establish an in vivo model of unilateral severe LIRI and to determine whether symptoms resembling PAGF after clinical lung transplantation could be induced. Although the use of warm rather than cold ischemia seems controversial, it has been demonstrated that there are no major differences between short periods of warm and longer periods of cold ischemia [15]. Moreover, warm ischemia has been used extensively in IRI models of liver and kidney as an accelerated model of clinically relevant cold IRI [16-19].

Since most studies have only investigated the early hours of reperfusion [19-32], the effect of severe LIRI up to months after reperfusion is unknown. Furthermore a detailed description of the subset of leukocytes and the time course of infiltration on both short and long term after LIRI is currently missing. Therefore, we have investigated a broad spectrum of LIRI parameters, including lung function, capillary permeability, matrix metallo proteinase (MMP) production, surfactant conversion, and histological changes on the short (days) and long-term (months) after LIRI and we have described leukocyte kinetics.

Finally, in the case of single lung transplantation, the changes in the native lung after transplantation of the contralateral side are not well established, especially on the long term. Therefore, we also assessed changes in non-ischemic right lung in animals undergoing left-sided LIRI.

## Methods

### Experimental design

The experimental protocol was approved by the Animal Experiments Committee under the Dutch National Experiments on Animals Act and complied with the 1986 directive 86/609/EC of the Council of Europe. Male Sprague-Dawley rats (n = 135, weighing 295 ± 4 grams) (Harlan, The Netherlands) were randomised into the experimental LIRI (n = 75), sham-operated (n = 50) or unoperated (n = 10) group. LIRI (n = 15 per time point) and sham-operated (n = 10 per time point) animals were killed on day 1, 3, 7, 30 or 90 postoperatively. Animals in the LIRI group were subjected to 120 minutes of warm ischemia of the left lung. Sham-operated animals underwent the same protocol as LIRI animals without applying left lung ischemia; unoperated controls were killed without any intervention.

### Surgical procedure

Animals were anesthetized with 60 mg/kg of ketaminhydrochloride intraperitoneally and a gas mixture (1.5–3% isoflurane, 57% NO_2 _and 40% O_2_), whereafter they were intubated and pressure control ventilated on a Siemens Servo 900C ventilator (Maquet Critical Care AB, Solna, Sweden) (14 cm H_2_O peak inspiratory pressure (PIP), 4 cm H_2_O positive end expiratory pressure (PEEP), frequency 40 breaths/minute, fraction of inspired oxygen (FiO_2_) 0.4). Following a left dorsolateral thoracotomy in the fourth intercostal space, the inferior pulmonary ligament was divided. The left lung was mobilized atraumatically, and lung ischemia was induced by clamping the bronchus, pulmonary artery and vein of the left inflated lung using a single noncrushing microvascular clamp. At reperfusion, the lung was recruited by a stepwise increase of PIP and PEEP (maximum respectively 50 and 18 cm H_2_O) until the lung was visually expanded. Recruitment was also performed in sham-operated animals. The thorax was closed and the animals received 5 ml of 5% glucose intraperitoneally and 0.1 mg/kg of buprenorphinhydrochloride (0.3 mg/ml) intramuscularly and were weaned from the ventilator. Body temperature was kept within normal range with a heating pad. All animals recovered with additional oxygen during the first 12 hours.

### Blood gas values

At the end of the experiment (at day 1, 3, 7, 30 or 90), animals were anesthetized with 20 mg/kg intraperitoneally administered pentobarbital (60 mg/ml) and a gas mixture (3% isoflurane, 64% NO_2 _and 33% O_2_). After weighing the animals, a polyethylene catheter (0.8 mm outer diameter) was inserted into the carotid artery and a metal cannula was inserted into the trachea. Thereafter, anesthesia was continued with 20 mg/kg pentobarbital intraperitoneally and 0.7 mg/kg pancuronium bromide (2 mg/ml) intramuscularly, whereafter animals were ventilated for 5 minutes (12 cm H_2_O PIP, 2 cm H_2_O PEEP, frequency 30 breaths/minute and FiO_2 _1.00). Blood gas values were recorded in 0.3 ml heparinized blood taken from the carotid artery (ABL555 gas analyzer, Radiometer, Copenhagen, Denmark). Animals were exsanguinated and euthanised by an overdose of pentobarbital (200 mg/kg), administered intravenously.

### Static compliance

The thorax and diaphragm were opened to eliminate the influence of chest wall compliance and abdominal pressure and a static pressure-volume curve (PVC) of the left and right lung together and left lung separately was recorded as described previously [33]. The PVC of the individual left lung was conducted by clamping the contralateral hilum. Maximal compliance (C_max_) was determined as the steepest part of the lung deflation curve. Maximal lung volume (V_max_), corrected for body weight, was recorded at a pressure of 35 cm H_2_O.

### Broncho-alveolar lavage

Left and right lung were lavaged separately five times with 5 ml sodium chloride containing 1.5 mM CaCl_2_. Total recovered volume of BALf was noted. Cell suspensions were centrifuged at 400 g and 4°C for 10 minutes to pellet the cells. Supernatant of BALf was taken and stored at -20°C for surfactant analysis and measurement of the amount of alveolar serum protein.

### Cell collection

Left and right lung, thoracic lymph nodes (TLN), and spleen were collected, smashed and suspended in NaCl. Cell suspensions were centrifuged at 400 g and 4°C for 10 minutes to pellet the cells. Red blood cells were lysed with erythrocyte lysis buffer, whereafter the suspension was washed with murine FACS buffer (MFB) (phosphate buffered saline (PBS), 0.05% weight/volume (w/v) sodium azide and 5% w/v bovine serum albumin (BSA)), centrifuged and resuspended in MFB. Cells were counted with a Bürker-Turk cell counter (Erma, Tokyo, Japan).

### Flow Cytometry

Pelleted cells (max 1*10^6 ^cells per well) were incubated on ice with 2% volume/volume (v/v) normal rat serum (NRS) in MFB for 15 minutes to prevent non-specific binding of Fc-receptors with primary antibodies. Hereafter, cells were washed, centrifuged and surface-stained for 30 minutes at 4°C in the dark with the following primary mouse anti rat antibodies: biotin conjugated CD5 (OX19^1^), phycoerythrin (PE) labelled CD8 (OX8^2^), fluorescein-isothiocyanate (FITC) labelled CD4 (OX38^2^), CD45RA-PE (OX33^1^), and HIS48^1^. After centrifuging and washing, primary staining of the HIS48 and OX-19-Biotin antibody was revealed by secondary staining with respectively goat anti mouse IgM, conjugated to PE (STAR86PE^1^) and streptavidin RPE-Cy5 (phycoerythrin-cychrome) (STAR89^1^) for 30 minutes at 4°C in the dark. Antibodies were obtained commercially from Serotec^1 ^(Kidlington, United Kingdom) and BD^2 ^(Franklin Lakes, New Jersey, USA).

Cellular differentiation was calculated based on morphology (Side SCatter (SSC) for granularity, Forward SCatter (FSC) for size), autofluorescence and specific positive antibody staining. Cells were identified as follows: Lymphocytes low FSC, low SSC, no autofluorescence, and expressing either CD45RA^+ ^(B-lymphocytes), CD5^+ ^(T-lymphocytes), CD5^+^CD4^+ ^(helper T-lymphocytes), and CD5^+^CD8^+ ^(cytotoxic T-lymphocytes); neutrophils low FSC, intermediate SSC and HIS48^+^; macrophages as high SSC and FSC and autofluorescent [34].

Data were acquired on a FACS Calibur flowcytometer (BD, Franklin Lakes, New Jersey, USA) and were analyzed using CellQuest (BD, Franklin Lakes, New Jersey, USA) and FlowJo software (Tree Star, Ashland, Oregon, USA).

### SA/LA ratio

Supernatant of BALf was centrifuged at 4°C for 15 minutes at 40.000 g to separate surface-active surfactant pellet (large aggregate (LA)) from a non-surface active supernatant fraction (small aggregate (SA)). LA was resuspended in 2 ml NaCl, whereafter the phosphorus concentration of LA and SA was determined by phospholipid extraction, followed by phosphorus analysis [35].

### Protein concentration

The supernatant was further used to determine alveolar protein concentration using the Bio-Rad protein assay (Bio-Rad, Hercules, California, USA) using a Beckmann DU 7400 photospectrometer with a wavelength set at 595 nm (Beckmann, Fullerton, California, USA) [36]. Bovine serum albumin was used as standard.

### Determination of matrix-metallo-proteinase activity

To determine the activity of MMP-2 and MMP-9, gelatin zymography was performed on BALf of the left lung (n = 6 per group, randomly assigned). Zymography was conducted on 10% SDS-polyacrylamide gels containing 1% w/v porcine skin gelatin (Sigma-Aldrich, St. Louis, Missouri, USA). The samples were 1:1 mixed with SDS-PAGE sample buffer (0.25 M Tris HCl, pH 6.8, 2% w/v SDS, 20% v/v glycerol, 0.01% v/v bromofenol blue), heated for 3 minutes at 55°C and subjected to standard electrophoretic analysis at room temperature using the protean II system (Bio-Rad, Hercules, California, USA). After electrophoresis, gels were washed two times for 15 minutes with 2.5% Triton X-100 buffer to renature MMPs by removal of SDS. Hereafter, gels were incubated with development buffer (5 mM CaCl_2_, 50 mM Tris HCl, pH 8.8, 0.02% w/v NaN_3_, aquadest) for 20 hours and proteins were fixated for 15 minutes using 45% v/v methanol and 10% v/v acetic acid. Gelatinolytic activity was visualized as clear zones after staining with 0.1% w/v Coomassie Brilliant Blue R-250 in 45% v/v methanol and 10% v/v acetic acid and subsequent destaining in the same solution without Coomassie Brilliant Blue. Gels were scanned (Kodak image station 440 cf; Kodak, Rochester, New York, USA) and quantified (Kodak image analysis software). A control sample was used in all gels to be able to compare the various blots. After measuring the band intensity of all blots, values were multiplied by a correction factor, determined by the values of the control sample.

### Histology

Histological assessment was performed in 3 animals per group per time point. The heart and lungs were excised en bloc, whereafter the lungs were fixated at a pressure of 10 cm H_2_O in 4% paraformaldehyde for 24 hours and embedded in paraffin wax. Sections were cut and stained with haematoxylin and eosin (HE). A histopathologist (MdB), blinded for the treatment, performed histological examination on the following parameters: intra-alveolar and septal edema, hyaline membrane formation, inflammation (classified as histiocytic, lymphocytic, granulocytic, and mixed), fibrosis, atelectasis, intra-alveolar hemorrhage, and overall classification. Each parameter was ranked as mild/scattered, moderate/occasional, or severe/frequent. Sections were overall classified as 1) normal, if no abnormalities were seen, 2) exsudative, if pulmonary edema and/or hyaline membranes were present, 3) fibroproliferative, if activated fibroblasts and/or proliferating alveolar type II cells were found, and 4) resolving, if injury was on return to normal.

Slides were scored on a Leica DMLB light microscope and photographes were taken using a Leica DC500 camera (Leica Microsystems AG, Wetzlar, Germany).

### Statistical analysis

The results in text, tables and figures are presented as mean ± standard error of the mean (SEM). Data were analysed using SPSS version 11.1 statistical software (SPSS Inc., Chicago, Illinois, USA). If an overall difference between groups was found by the Kruskal-Wallis test, Mann-Whitney U tests were performed for intergroup comparison. Difference in mortality rate was assessed by the Fisher's exact test. P values < 0.05 were considered to be significant.

## Results

### Survival and weight loss

All sham-operated animals survived the experimental period. LIRI resulted in a mortality rate of 25% (0/50 in sham-operated animals versus 19/75 after LIRI, *P *< 0.0001). Non-surviving LIRI animals died shortly after weaning due to the development of pulmonary edema. Surviving LIRI animals had lost more weight on day 3 as compared to sham-operated rats (-34.91 ± 3.86 g versus -21.10 ± 2.86 g, *P *= 0.01). From day 7 on these differences had disappeared.

### PaO_2 _& PaCO_2_

Arterial oxygenation was lower in LIRI animals than in unoperated and sham-operated controls on day 1, 3, and 7 (Table 1). On day 30 and 90, these differences had disappeared. An elevated PaCO_2 _was found 1 day after LIRI, as compared to unoperated and sham-operated animals.

**Table 1:** PaO_2_/FiO_2 _and PaCO_2_/FiO_2 _ratio, based on both lungs

**Blood gas values**	**Mean PaO_2_/FiO_2 _(SEM) [mm Hg]**	**Mean PaCO_2_/FiO_2 _(SEM) [mm Hg]**
Unoperated	562 (25)	45.4 (4.6)
Sham day 1	559 (17)	39.3 (2.3)
Sham day 3	520 (23)	40.8 (4.5)
Sham day 7	573 (17)	50.4 (7.8)
Sham day 30	561 (12)	41.5 (3.5)
Sham day 90	576 (21)	37.2 (4.0)
LIRI day 1	282 (41) **US**^1^**L**^7–90^	61.1 (6.1) **US**^1^**L**^7–90^
LIRI day 3	241 (38)**US**^3^**L**^7–90^	48.0 (4.8)
LIRI day 7	435 (48)**US**^7^**L**^90^	44.8 (2.2)
LIRI day 30	543 (22)	42.3 (2.0)
LIRI day 90	607 (14)	30.2 (2.4) **UL**^1–30^

U = P < 0.05 versus unoperated animals

S^x-y ^= P < 0.05 versus sham-operated animals from day x until day y

L^x-y ^= P < 0.05 versus LIRI animals from day x until day y

FiO_2 _= Fraction of inspired Oxygen; LIRI = Lung Ischemia-Reperfusion Injury; PaO_2 _= Arterial Oxygen pressure; PaCO_2 _= Arterial Carbon dioxide pressure; SEM = Standard-Error of the Mean

### Static compliance of the left lung

LIRI had detrimental effects on both the Cmax and Vmax of the left ischemic lung as compared to control lungs (Table 2). Up to 90 days after LIRI, Vmax and Cmax of the left lung remained lower than in sham-operated and unoperated rats.

**Table 2:** Static compliance of the left lung, corrected for body weight

**Left Lung Compliance**	**Mean Vmax (SEM) [ml/kg]**	**Mean Cmax (SEM) [(ml/kg)/cm H**_2_**O]**
Unoperated	13.4 (0.48)	1.12 (0.10)
Sham day 1	15.9 (1.13)	1.32 (0.11)
Sham day 3	15.9 (0.81) **U**	1.26 (0.18)
Sham day 7	14.1 (1.21)	0.95 (0.04) **S**^1^
Sham day 30	12.3 (0.63) **S**^1–3^	1.00 (0.08) **S**^1^
Sham day 90	11.8 (0.58) **S**^1–3^	1.09 (0.06)
LIRI day 1	4.8 (0.59) **US**^1^**L**^7^	0.29 (0.05)**US**^1^**L**^30–90^
LIRI day 3	5.0 (0.68) **US**^3^**L**^7^	0.32 (0.05) **US**^3^**L**^90^
LIRI day 7	9.0 (1.51) **US**^7^	0.53 (0.12) **US**^7^
LIRI day 30	6.2 (0.75) **US**^30^	0.51 (0.06) **US**^30^
LIRI day 90	6.9 (1.04) **US**^90^	0.67 (0.11) **US**^90^

U = P < 0.05 versus unoperated animals

S^x-y ^= P < 0.05 versus sham-operated animals from day x until day y

L^x-y ^= P < 0.05 versus LIRI animals from day x until day y

Cmax = Maximal compliance of the expiration curve, corrected for body weight; LIRI = Lung Ischemia-Reperfusion Injury; SEM = Standard-Error of the Mean; Vmax = Maximal lung volume corrected for body weight at a pressure of 35 cm H_2_O

### Capillary permeability

The alveolar serum protein level of the ischemic left lung, as parameter for capillary permeability, was increased 1 day after reperfusion as compared to controls (Table 3). On day 3 the amount of alveolar serum protein in left BALf of LIRI animals was still higher than in unoperated rats. From day seven on, no differences were present.

**Table 3:** Alveolar serum proteins of the left lung

**Alveolar proteins**	**Mean Proteins (SEM) [μg/ml]**
Unoperated	226 (51)
Sham day 1	386 (131)
Sham day 3	323 (76)
Sham day 7	154 (51)
Sham day 30	151 (50)
Sham day 90	202 (65)
LIRI day 1	1,663 (202) **US**^1^**L**^3–90^
LIRI day 3	447 (75) **UL**^7–90^
LIRI day 7	168 (60)
LIRI day 30	79 (25)
LIRI day 90	74 (25)

U = P < 0.05 versus unoperated animals

S^x-y ^= P < 0.05 versus sham-operated animals from day x until day y

L^x-y ^= P < 0.05 versus LIRI animals from day x until day y

LIRI = Lung Ischemia-Reperfusion Injury; SEM = Standard-Error of the Mean.

### Matrix metalloproteinase activity

**Figure 1:**
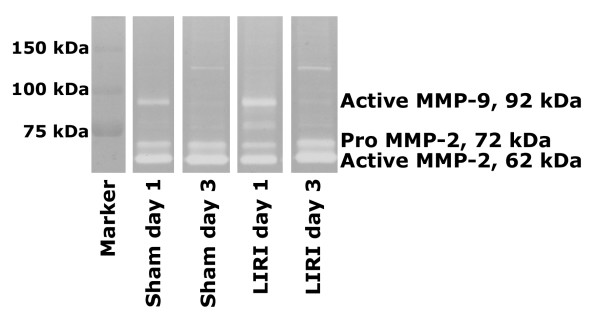
MMP-2 and MMP-9 zymography. Pro MMP-9 was not measurable in any of the samples and active MMP-9 was detectable in the BALf of sham-operated and LIRI animals on day 1. Pro and active MMP-2 is expressed constitutively in all animals. BALf = Broncho-Alveolar Lavage Fluid; LIRI = Lung Ischemia-Reperfusion Injury; MMP = Matrix MetalloProteinase.

**Figure 2:**
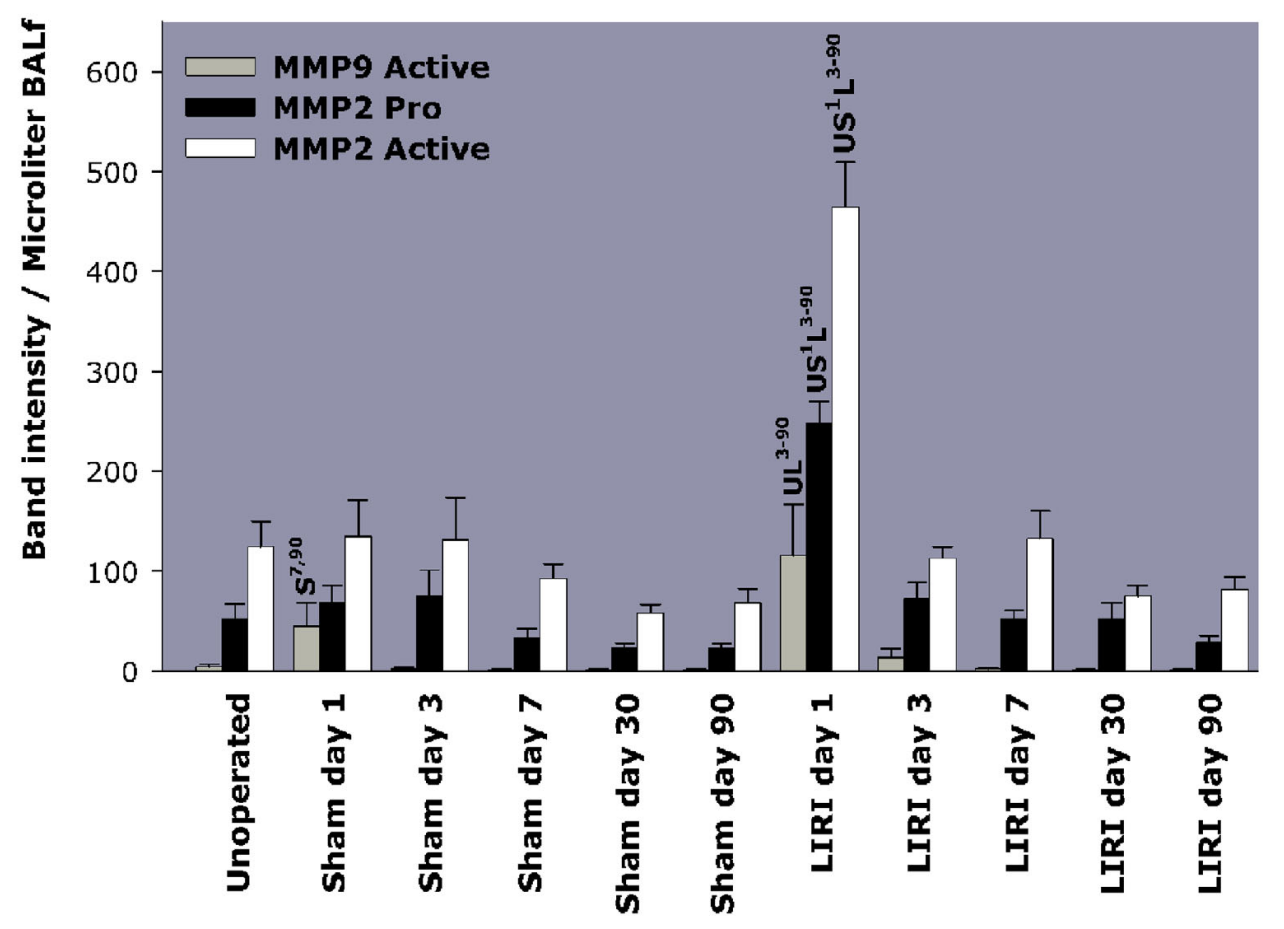
MMP production measured in BALf by zymography. On day 1, significant more pro-, and active MMP-2 and active MMP-9 was found in the BALf of LIRI animals as compared to sham-operated and unoperated controls. BALf = Broncho-Alveolar Lavage Fluid; LIRI = Lung Ischemia-Reperfusion Injury; MMP = Matrix MetalloProteinase. U = P < 0.05 versus unoperated animals. S^x-y ^= P < 0.05 versus sham-operated animals from day x until day y. L^x-y ^= P < 0.05 versus LIRI animals from day x until day y

MMP-2 is expressed constitutively in all animals (Figure 1 and 2). However, the total amount of pro- and active MMP-2 and MMP-9 per microliter BALf is increased in LIRI animals on day 1 (Figure 2) (recovered volume did not differ between the groups). MMP activity per microgram protein in the BALf, does not differ between the groups (data not shown), which indicates that the increased activity after LIRI must be due to elevated alveolar serum proteins. After day 3, no differences were demonstrable between the groups.

### Surfactant small and large aggregates

**Figure 3:**
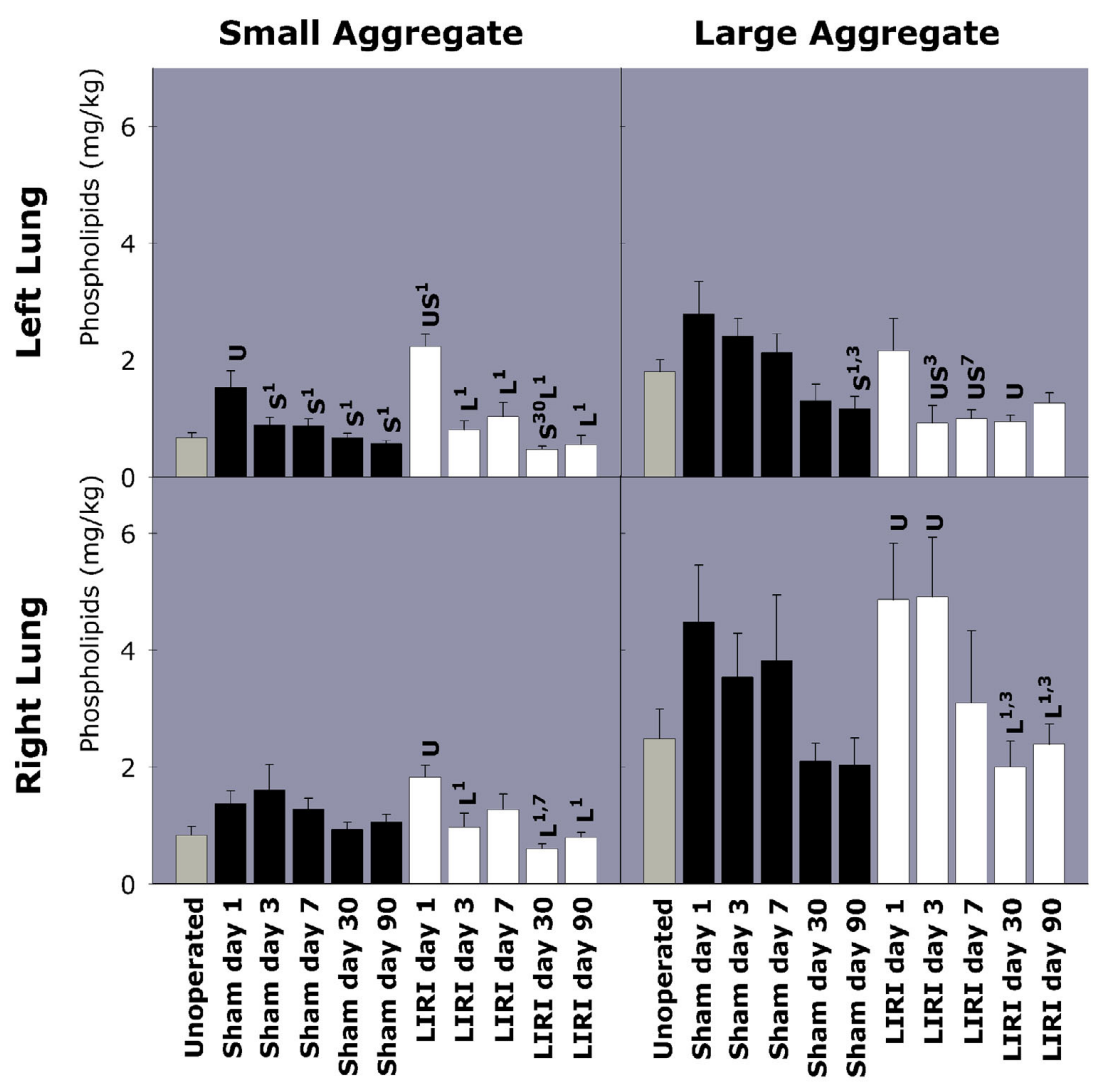
Total amount of SA and LA phospholipids in left and right BALf. SA and LA phospholipids (mg/kg body weight) were measured in left and right BALf of unoperated, sham-operated and LIRI animals on day 1, 3, 7, 30 and 90. Elevated levels of SA were found in both left and right BALf on day 1 and a decreased level of LA was measured up to day 30 in LIRI animals. BALf = Broncho-Alveolar Lavage Fluid; LIRI = Lung Ischemia-Reperfusion Injury; SA = Small Aggregate; LA = Large Aggregate. U = P < 0.05 versus unoperated animals. S^x-y ^= P < 0.05 versus sham-operated animals from day x until day y. L^x-y ^= P < 0.05 versus LIRI animals from day x until day y

While an increase in SA was found in the BALf of the left lung of sham-operated animals on day 1, a higher level was measured in LIRI lungs (Figure 3). After LIRI, an elevated amount of SA was also found in the right lung on day 1. The amount of LA in the left lung was decreased from day 3 until day 30 following LIRI, whereafter the LA level returned to normal on day 90.

### Infiltrating cells

#### Neutrophils

**Figure 4:**
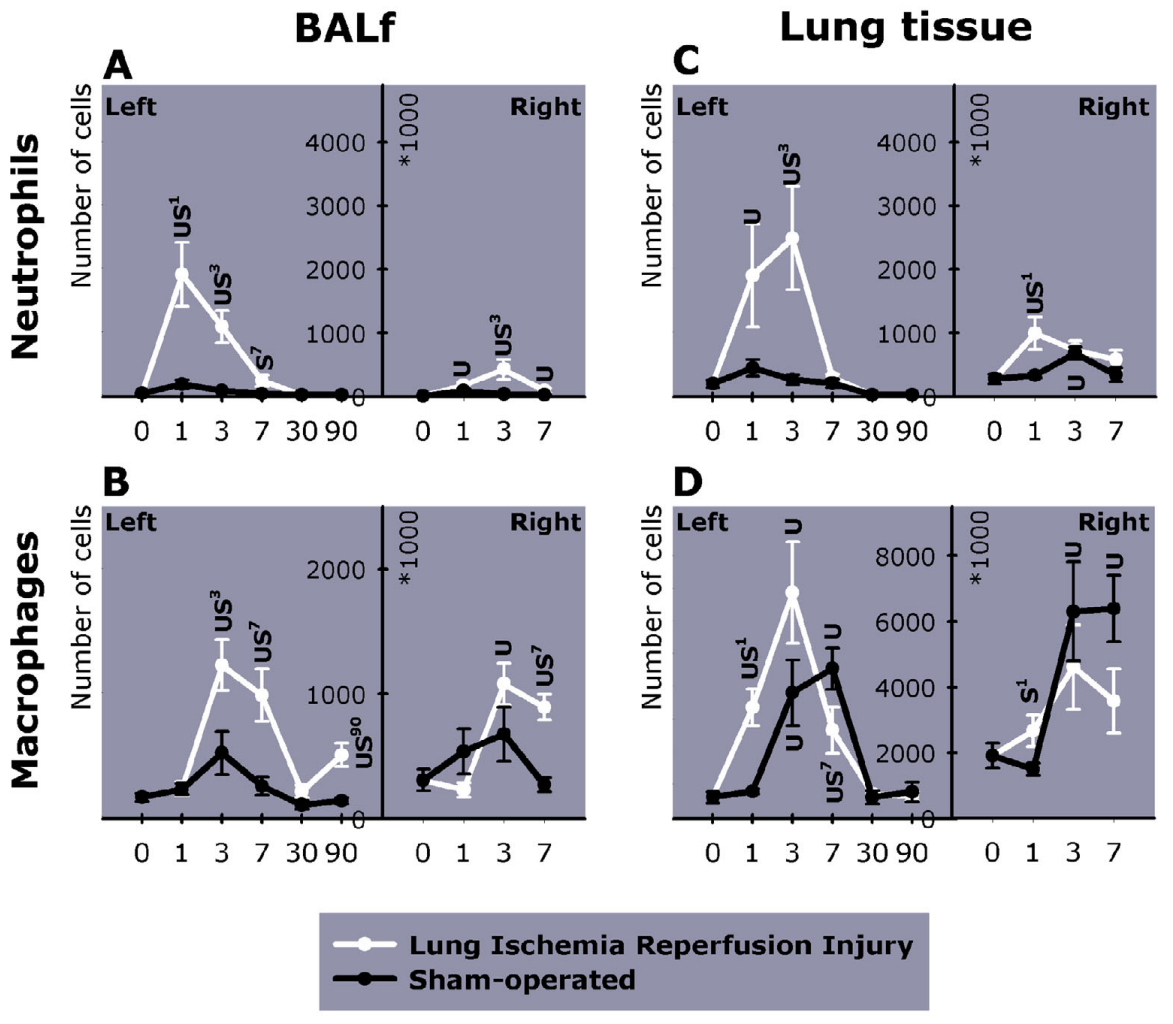
The number of inflammatory cells in BALf and lung tissue of the left (day 0–90) and right lung (day 0–7). Shown are (A) neutrophils, and (B) macrophages in BALf; (C) neutrophils, and (D) macrophages in lung tissue. Day 0 represents the baseline value measured in unoperated animals. BALf = Broncho-Alveolar Lavage Fluid. U = P < 0.05 versus unoperated animals. S^x-y ^= P < 0.05 versus sham-operated animals from day x until day y. L^x-y ^= P < 0.05 versus LIRI animals from day x until day y

Sham operation resulted in some infiltration of neutrophils in the first days after the operation, as demonstrated by an elevated percentage in left and right BALf and lung tissue (see additional file 1, Table 4A, 5A, 6A and 7A). However, after LIRI even more neutrophils were measured in predominantly the left, but also the right BALf (Figure 4A this manuscript; see additional file 1, Table 4B and 5B) and lung tissue (Figure 4C this manuscript; see additional file 1, Table 6B and 7B). Hereafter the number of neutrophils gradually decreased, and could not be measured anymore on days 30 and 90.

#### Macrophages

Macrophage occurrence followed similar kinetics in sham-operated and ischemic lungs, but more macrophages were present on day 1 and 3 in ischemic lung tissue and on day 3 and 7 in BALf (Figure 4B and 4D this manuscript; see additional file 1, Table 4B and 6B). LIRI also led to an increase in macrophages in the BALf of the contralateral lung on day 3 and 7 as compared to sham and unoperated animals (Figure 4B this manuscript; see additional file 1, Table 5B). Although in sham-operated and LIRI animals macrophages had returned to normal on day 30 in left BALf, they were again elevated on day 90 (Figure 4B this manuscript, see additional file 1, Table 4B).

#### Lymphocytes

**Figure 5:**
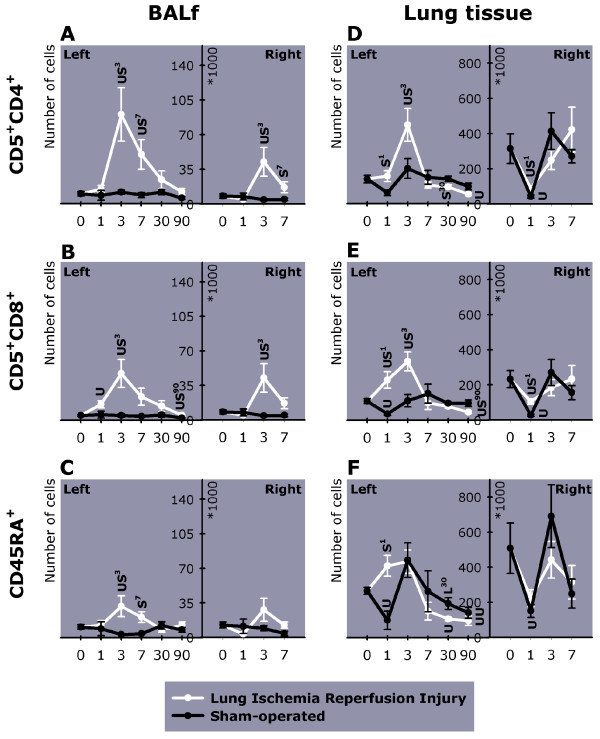
The number of inflammatory cells in BALf and lung tissue of the left (day 0–90) and right lung (day 0–7). Shown are (A) helper T-lymphocytes (CD5^+^CD4^+^), (B) cytotoxic T-lymphocytes (CD5^+^CD8^+^), and (C) B-lymphocytes (CD45RA^+^) in BALf; (D) helper T-lymphocytes, (E) cytotoxic T-lymphocytes, and (F) B-lymphocytes in lung tissue. Day 0 represents the baseline value measured in unoperated animals. BALf = Broncho-Alveolar Lavage Fluid. U = P < 0.05 versus unoperated animals. S^x-y ^= P < 0.05 versus sham-operated animals from day x until day y. L^x-y ^= P < 0.05 versus LIRI animals from day x until day y

Sham operation did not result in infiltration of lymphocytes in BALf (Figure 5A–C this manuscript; see additional file 1, Table 4B). After LIRI, an infiltration of mainly CD5^+^CD4^+ ^and CD5^+^CD8^+ ^and to a lesser extent CD45RA^+^-lymphocytes occurred in mainly the left, but also right BALf. Lymphocyte infiltration peaked on day 3, with levels decreasing thereafter (Figure 5A–C this manuscript; see additional file 1, Table 4B and 5B).

Although lymphocytes in right lung tissue of LIRI animals followed the same kinetics as in sham-operated animals, demonstrated by a decreased number on day 1 (Figure 5D–F this manuscript; see additional file 1, Table 7B), more CD5^+^CD4^+ ^and CD5^+^CD8^+^-cells were found in left lung tissue on day 1 and 3 as compared to sham-operated and unoperated animals (Figure 5D–E this manuscript; see additional file 1, Table 6B). On day 1 also more CD45RA^+^-cells were present in the left lung of LIRI animals (Figure 5F this manuscript; additional file 1, Table 6B). On day 90, the level of CD5^+^CD4^+^, CD5^+^CD8^+^, and CD45RA^+ ^lymphocytes in left lung tissue of LIRI animals had decreased as compared to controls (Figure 5D–F this manuscript; see additional file 1, Table 6B).

**Figure 6:**
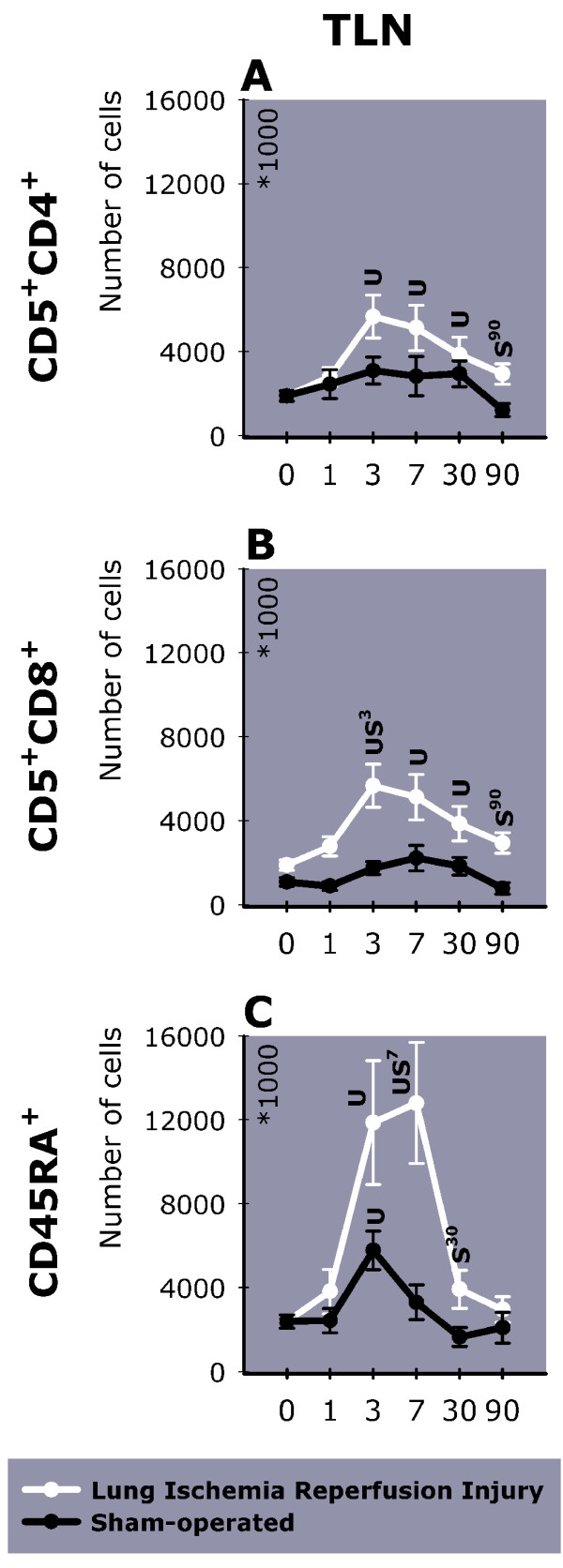
The number of inflammatory cells in TLN (day 0–90). Shown are (A) helper T-lymphocytes (CD5^+^CD4^+^), (B) cytotoxic T-lymphocytes (CD5^+^CD8^+^), and (C) B-lymphocytes (CD45RA^+^) in TLN. Day 0 represents the baseline value measured in unoperated animals. TLN = Thoracic Lymph Nodes; U = P < 0.05 versus unoperated animals. S^x-y ^= P < 0.05 versus sham-operated animals from day x until day y. L^x-y ^= P < 0.05 versus LIRI animals from day x until day y

No differences were found between groups in percentage or total number of cells within the spleen (data not shown). However, more CD5^+^CD4^+^, and CD5^+^CD8^+^-cells were measured in TLN on day 3 (Figure 6A–C this manuscript; see additional file 1, Table 8B). Whereas CD5^+^CD4^+ ^and CD5^+^CD8^+^-cells remained higher in LIRI animals than in unoperated animals up to day 90, CD45RA^+^-cells had returned to preoperative values on day 90.

### Histology

**Figure 7:**
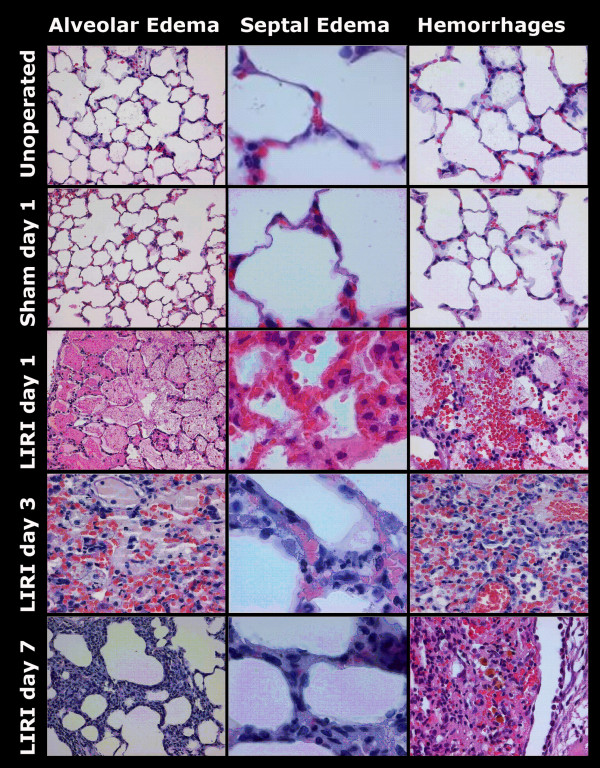
Histological examples of alveolar edema (25*), septal edema (100*) and intra-alveolar hemorrhage (40*) on HE slides. LIRI caused alveolar and septal edema and alveolar hemorrhages, which were most severe on day 1 and 3 after LIRI and resolved thereafter. On day 7 brownish macrophages were found after clearance of erythrocytes in the alveolus. HE = Haematoxylin and Eosin staining; LIRI = Lung Ischemia-Reperfusion Injury.

**Figure 8:**
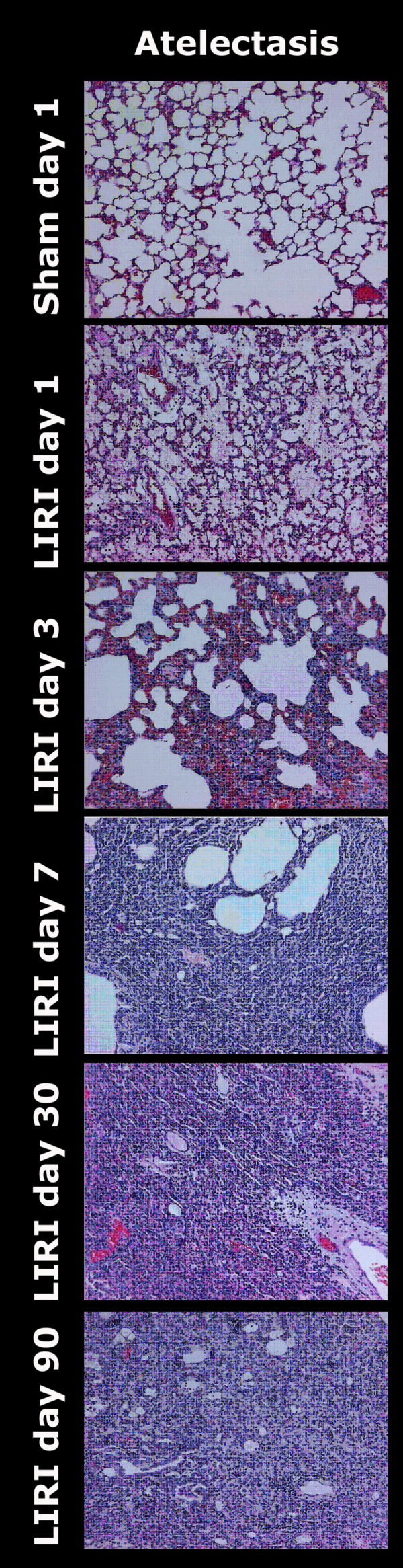
Histological examples of atelectasis (10*) on HE slides. Severe atelectasis was demonstrated up to day 90 after LIRI. HE = Haematoxylin and Eosin staining; LIRI = Lung Ischemia-Reperfusion Injury.

**Figure 9:**
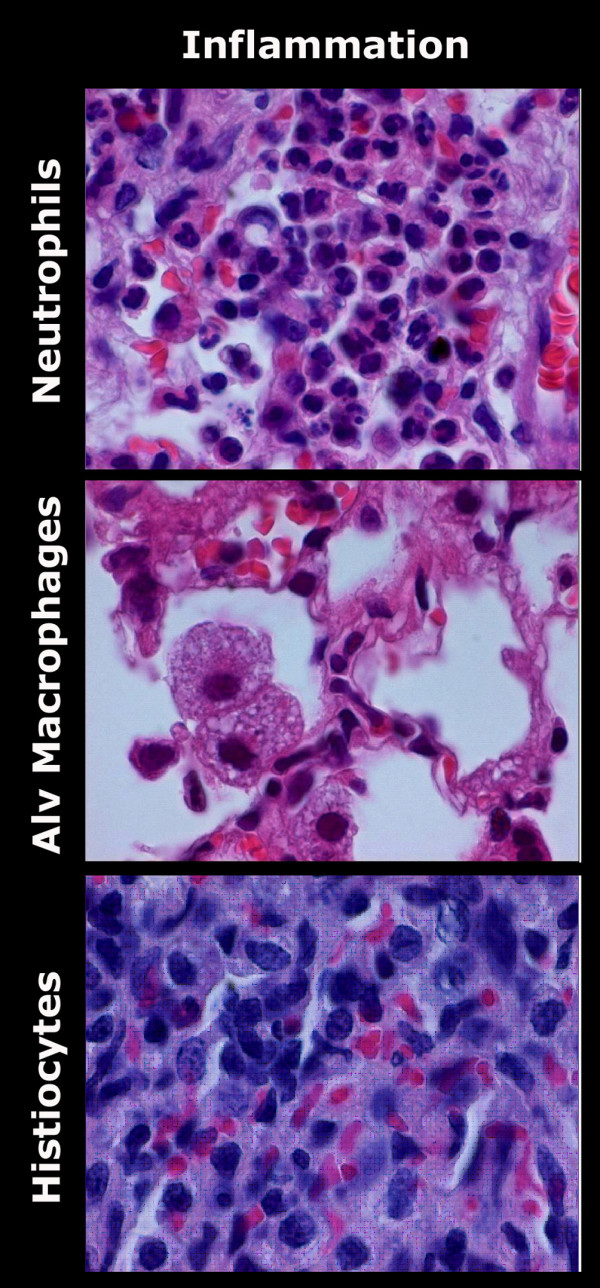
Histological examples of the inflammatory pattern of LIRI on HE slides (100*). Histological analysis confirms the flowcytometric analysis with the presence of predominantly neutrophils on day 1, alveolar macrophages on day 3, and histiocytes on day 30 following LIRI. HE = Haematoxylin and Eosin staining; LIRI = Lung Ischemia-Reperfusion Injury

LIRI resulted in diffuse alveolar damage consisting of severe intra-alveolar edema up to day 3, septal edema, which was mild on day 1 and increased to moderate on day 3, and intra-alveolar hemorrhages (Figure 7 this manuscript; see additional file 1, Table 9). The overall classification of LIRI animals changed from exsudative on day 1 to proliferative from day 3 to day 90. Although no atelectasis and fibrosis were seen on day 1 following LIRI, mild fibrosis and mild to severe atelectasis were seen from day 3 up to day 90 after LIRI (Figure 8 this manuscript; see additional file 1, Table 9). Identification of infiltrating cells confirmed the flowcytometry measurements. A mild inflammatory pattern consisting of histiocytes was found on day 3 and 7 in sham-operated animals. LIRI caused moderate to severe inflammation, which changed from mixed (granulocytic, lymphocytic, and histiocytic) inflammation on day 1 to a histiocytic and lymphocytic pattern from day 3 to 90 (Figure 9 this manuscript; see additional file 1, Table 10). No major differences between unoperated, sham-operated and LIRI animals were found in the right lung (data not shown).

## Discussion

This study describes the effect of warm LIRI on a broad spectrum of LIRI parameters, such as lung function, capillary permeability, MMP production, surfactant conversion, and histology on the short and long term after LIRI. Furthermore, a detailed description of the subsets of leukocytes and the time course of infiltration on both short and long term after LIRI is given.

LIRI has been suggested to be a major risk factor for PAGF. The clinical course of PAGF symptomatically resembles the acute respiratory distress syndrome (ARDS) and can be characterized by different stages, each with their specific clinical, histological and immunological changes [37]. The acute, exsudative phase is featured by a sudden onset of hypoxemia, decreased lung compliance, increased pulmonary artery pressure, and development of non-cardiogenic pulmonary edema [37,38]. Experimental studies have shown that abnormalities in, and depletion of pulmonary surfactant contribute to these symptoms of LIRI [26-28,39-41]. Histological analysis of LIRI shows diffuse alveolar damage with atelectasis, inflammation, intra-alveolar hemorrhage, formation of hyaline membranes and protein-rich edema [1,37]. Finally, production of matrix-metalloproteinases (MMP) is thought to be important in the acute phase of PAGF since MMPs increase the microvascular permeability and thereby enable extravasation of inflammatory cells [42-45].

We found hypoxemia, impaired left lung compliance, a mortality rate of 25% due to development of severe pulmonary edema, and an increase in SA subtype surfactant as early effects of LIRI. Conversion of highly surface active LA into poor surface active SA occurs shortly after reperfusion and is partly due to increased capillary permeability, resulting in influx of serum proteins into the alveolus, as confirmed in our study [28,46]. Serum proteins inhibit surfactant in a dose-dependent manner by competing with surfactant components at the alveolo-capillary barrier [47]. HE slides confirmed extensive alveolar and septal edema, intra-alveolar bleeding, atelectasis and inflammation, which are all indicative of diffuse alveolar damage. The increase in alveolar proteins and neutrophils on day 1 occurred simultaneously with an increased MMP activity. MMP-2 is usually constitutively expressed by endothelial cells, vascular smooth muscle cells and fibroblasts, fitting with the observation in our study of high levels of both pro-, and active MMP-2 found in unoperated animals [42-45,48]. Yano et al demonstrated that LIRI resulted in MMP-9 induction, but not MMP-2 expression 24 hours after LIRI [45]. A higher concentration of MMP-9 was also found in lung edema fluid of ARDS patients [49]. Nevertheless, we found that levels of both pro- and active MMP-9 and MMP-2 per microliter BALf are elevated 24 hours after LIRI, which correlates well with the presence of neutrophils in left lung BALf. Thus 120 minutes of warm ischemia resulted in our experimental study in a mortality rate of 25%, hypoxemia, early impaired left lung compliance, surfactant conversion, diffuse alveolar damage on HE slides and MMP production, which are all features of the exsudative phase of PAGF.

Human lung transplant patients surviving the acute phase of LIRI may either recover from injury or enter a 'chronic' fibroproliferative state, which develops within 4–7 days after the onset of symptoms [37,38]. The progression from an exsudative phase to a 'chronic' fibroproliferative state within one week after LIRI is supported in our study by the presence of fibroproliferative changes on HE slides in the first week after LIRI and an increased number of macrophages, which are important mediators in the regulation of fibroblast function. Importantly, LIRI induced progressive changes resulting in extensive pulmonary injury up to 3 months after reperfusion. This is demonstrated by a decreased number of lymphocytes found in lung tissue on day 30 and 90, impaired left lung compliance up to day 90, extensive atelectasis on HE slides, and a decreased surfactant recycling and secreting capacity of alveolar type II cells reflected by the decreased LA surfactant subtype [50]. Although extensive left pulmonary injury was found on the long-term, hypoxemia was demonstrated up to day 7. Thereafter, no differences in PaO_2 _were measured between LIRI animals and controls. This discrepancy may be explained by the fact that PaO_2 _was dependent on both lungs, so that the loss of left lung function was compensated by the right lung. Furthermore, even though MMPs are important mediators of pulmonary remodeling, no changes in activity on the long-term were found. It is questionable however whether MMPs are present in the BALf of severe atelectatic and fibrotic lungs. Therefore, in future studies, measurement of MMP activity should also be performed in homogenized lung tissue.

Thus, 120 minutes of warm ischemia in this model induces injury comparable to PAGF and ARDS in clinical lung transplantation on the short, but also on the long-term. Nevertheless, this experimental model has several shortcomings we wish to address. First of all, we used warm ischemia to induce LIRI, whereas in the clinical setting cold ischemic time is associated with PAGF. However, it has been demonstrated that there are no major differences between short periods of warm and longer periods of cold ischemia and warm ischemia has been used extensively in IRI models of liver and kidney as an accelerated model of clinically relevant cold IRI [15-19]. Another disadvantage is that a rather long period of 120 minutes warm ischemia has been used. Shorter periods of warm ischemia have been investigated in a pilot study to setup our model (data not shown) and we found that 120 minutes of warm ischemia is necessary in our hands to induce symptoms comparable to PAGF. This finding is supported by a clinical study by Thabut et al, which shows that the relationship between cold graft ischemic time and survival appears to be of cubic form with a cutoff value of 330 minutes [3]. Thereafter short-term mortality increases rapidly mainly due to development of PAGF [3].

Another goal of this study was to describe leukocyte kinetics following LIRI. The immunologic effects of LIRI have only been studied up to hours after reperfusion, whereas we investigated leukocyte kinetics after LIRI up to 90 days post-reperfusion. Several studies have shown that macrophages are activated during ischemia, followed by the recruitment of neutrophils within hours after the start of reperfusion [16,19,20,22,24,25,29,51,52]. We now add that neutrophil infiltration lasted for 3 days after reperfusion, thereby strengthening the theory that neutrophils are important in perpetuating LIRI. The extended presence of neutrophils after LIRI may be also explained by the fact that phagocytes are important elements of the repair process after LIRI by clearing apoptotic cells and necrotic debris [29]. Nevertheless, since ischemia-reperfusion injury still develops in neutropenic models, it is questionable whether neutrophils are pivotal in LIRI [21,32].

In this regard, other studies suggest an early role for T cells as important mediators of ischemia induced injury. An infiltration of CD4^+^-T-cells occurred in these studies from 1 until 12 hours after reperfusion and disappeared hereafter [16,19,20,51], whereas we found elevated numbers of mainly CD5^+^CD4^+ ^and CD5^+^CD8^+ ^T-lymphocytes 3 and 7 days after reperfusion in the left lung and TLN. Infiltration and activation of T cells has been classically attributed to the presence of antigen; our findings in an autologous setting may be explained by different mechanisms. First, LIRI induced upregulation of adhesion molecules may attract T cells, such as effector and memory T-cells, which are able to proliferate in an antigen independent fashion by cytokines produced locally (bystander effect), in contrast with naïve T-cells which require antigen presentation by antigen presenting cells [53]. Moreover, antigen specific T-cells may be attracted by released self-antigens [54,55]. Myosin, heat shock proteins and type V collagen, which is released after LIRI in BALf comparable to the level observed in allografted lungs, have been shown to be capable of inducing a T-helper-cell reaction within days after reperfusion [54,55]. The latter is supported by the finding of Waddell and colleagues, who demonstrated upregulation of major histocompatibility II complex after LIRI [56]. Finally, the elevated levels of CD5^+^CD4^+ ^and CD5^+^CD8^+ ^may be explained by their possible role in the pathogenesis of lung fibrosis, which is also supported by the presence of macrophages in the BALf and lung parenchyma of ischemic animals 3, 7, and 90 days after reperfusion, since they are also thought to be important mediators in the regulation of fibroblast function [37,38,57,58].

Our study furthermore demonstrates an immunosuppressive effect of operation, as measured by the decreased number of lymphocytes in lung tissue on day 1. Although it is very well known that major surgery may cause a short-lasting decrease in blood circulating lymphocytes [59], we now additionally report that thoracotomy causes a one day decrease in the number of lymphocyte subset in lung parenchyma, while the number of lymphocytes in the BALf of sham-operated animals is close to normal. The immunosuppressive effect of surgery may be due to reduced T-cell proliferation and reduced secretion of interleukin-2 (IL-2), IL-4, and gamma interferon by T-lymphocytes, which may be the effect of inhibitory factors secreted by mononuclear phagocytic cells as a result of injury [60]. Moreover, altered migration of memory and activated effector T cells to injury sites may have also contributed to the decreased level of measured cells [61].

Finally, another interesting point arising from this study is the effect of left LIRI on right-sided pulmonary injury. While no major changes were seen on HE slides of the right lung, the inflammatory profile of the right BALf resembled that of the left, although it was less severe. Also, an increased amount of SA was measured in these parts of the lung, demonstrating that the right lung has sustained injury. Since the right lung did not sustain ischemia, induction of systemic components, similar to that seen after mesenteric artery ischemia, may have caused right lung injury. In this regard, induction of high-mobility group-1 protein, a downstream proinflammatory cytokine produced by necrotic cells [62,63], and production of uric acid could explain this phenomenon [64]. Furthermore, activated neutrophils lose their ability to deform, so that they might have plugged the capillaries of the right lung and may have subsequently caused lung injury [65]. However, LIRI of the left lung did not result in long-term damage in the right lung.

## Conclusion

The short and long-term changes after LIRI in this model resemble those found in both PAGF and ARDS after clinical lung transplantation. Thus LIRI seems a major risk factor for PAGF in the absence of other influencing factors, such as alloimmunity. Importantly, LIRI resulted in progressive deterioration of lung function and architecture, leading to extensive immunopathological and functional abnormalities up to 3 months after reperfusion. Immunologically, LIRI caused neutrophil infiltration early after reperfusion, followed by T lymphocytes and macrophages. The non-ischemic lung also showed signs of inflammation on the short-term, but to a lesser extend, and long-term changes were not found in the right lung.

## Abbreviations

ARDS: Acute Respiratory Distress Syndrome; BALf: Broncho Alveolar Lavage Fluid; BSA: Bovine Serum Albumine; Cmax: Maximal Compliance of the expiration curve; FACS: Fluorescence Activated Cell Sorter; FiO_2:_ Fraction of inspired Oxygen; FITC: Fluorescein-IsoThioCyanate; FSC: Forward Scatter; HE: Haematoxylin and Eosin; IL: InterLeukin; LA: Large Aggregate surfactant subtype; LIRI: Lung Ischemia Reperfusion Injury; MFB: Murine FACS Buffer; MMP: Matrix Metallo Proteinase; NRS: Normal Rat Serum; PAGF: Primary Acute Graft Failure; PaO_2:_ Arterial Oxygen Pressure; PBS: Phosphate Buffered Saline; PE: PhycoErythrin; PE-Cy5: PhycoErythrin-Cychrome 5; PEEP: Positive End Expiratory Pressure; PIP: Peak Inspiratory Pressure; PVC: Pressure Volume Curve; SA: Small Aggregate surfactant subtype; SSC: Side Scatter; TLN: Thoracic Lymph Nodes; V/V: Volume/Volume; Vmax: Maximal Lung Volume at a pressure of 35 cm H_2_O; W/V: Weight/Volume.

## Competing interests

The author(s) declare that they have no competing interests.

## Authors' contributions

All authors were involved in the experimental design, interpretation of the data and in the preparation of this manuscript. Furthermore, all authors read and approved the final manuscript. NPvdK operated the animals, collected and analyzed the data, and prepared the manuscript. JK and AJJCB participated in the cardiothoracic approach of this model. JJH and BL took care of the anaesthetic part of this model and performed the surfactant and protein analysis of the supernatant. MAdB performed all histological analysis. BNL contributed to the immunological analysis of LIRI and was of essential help in analysis of the FACS data. RWFdB performed all MMP measurements.

## Supplementary Material

Additional file 1 Table 4A, 4B, 5A, 5B, 6A, 6B, 7A, 7B, 8A, 8B, 9, 10. Table 4A: % of inflammatory cells in left BALf. Table 4B: Total number of inflammatory cells in left BALf. Table 5A: % of inflammatory cells in right BALf. Table 5B: Total number of inflammatory cells in right BALf. Table 6A: % of inflammatory cells in left lung tissue. Table 6B: Total number of inflammatory cells in left lung tissue. Table 7A: % of inflammatory cells in right lung tissue. Table 7B: Total number of inflammatory cells in right lung tissue. Table 8A: % of inflammatory cells in TLN. Table 8B: Total number of inflammatory cells in TLN. Table 9: Histologic general score of the left lung. Table 10: Histologic inflammatory score of the left lung Click here for file
