# Supplementary material for: Ischemia of the lung causes extensive long-term pulmonary injury: an experimental study
Source: Respir Res. 2008 Mar 26;9(1):28. doi: 10.1186/1465-9921-9-28 (PMC2335107; doi:10.1186/1465-9921-9-28)
Supplement: Additional file 1 — Table 4A, 4B, 5A, 5B, 6A, 6B, 7A, 7B, 8A, 8B, 9, 10. Table 4A: % of inflammatory cells in left BALf. Table 4B: Total number of inflammatory cells in left BALf. Table 5A: % of inflammatory cells in right BALf. Table 5B: Total number of inflammatory cells in right BALf. Table 6A: % of inflammatory cells in left lung tissue. Table 6B: Total number of inflammatory cells in left lung tissue. Table 7A: % of inflammatory cells in right lung tissue. Table 7B: Total number of inflammatory cells in right lung tissue. Table 8A: % of inflammatory cells in TLN. Table 8B: Total number of inflammatory cells in TLN. Table 9: Histologic general score of the left lung. Table 10: Histologic inflammatory score of the left lung [file 1465-9921-9-28-S1.doc]

**Additional data files**

**Table 4A. The percentage of inflammatory cells (mean (SEM)) in left broncho-alveolar lavage fluid.**

| **Left BALf** | **% Cells** | | | | |
| --- | --- | --- | --- | --- | --- |
|  | **N** | **M** | **CD4** | **CD8** | **CD45** |
| **Unoperated** | 3.5  (0.9) | 31  (5.2) | 2.0  (0.4) | 1.0  (0.2) | 2.1  (0.4) |
| **Sham day 1** | 24 **US^30-90^**  (8.4) | 53  (9.3) | 0.4 **US^7-90^**  (0.1) | 0.2 **US^7-30^**  (0.1) | 0.3 **US^30-90^**  (0.1) |
| **Sham day 3** | 11 **S^30-90^**  (2.7) | 47  (7.1) | 1.1  (0.4) | 0.4  (0.2) | 0.5 **US^30-90^**  (0.1) |
| **Sham day 7** | 6.1 **S^30-90^**  (1.9) | 52 **US^30-90^**  (4.4) | 1.8  (0.3) | 0.9  (0.2) | 1.2  (0.4) |
| **Sham day 30** | ND | 23  (7.5) | 2.9  (0.7) | 1.1  (0.5) | 3.0  (0.7) |
| **Sham day 90** | ND | 34  (4.2) | 1.7  (0.4) | 0.4 **U**  (0.0) | 2.0  (0.7) |
| **LIRI day 1** | 45 **UL^7-90^**  (8.3) | 13 **US^1^L^3-90^**  (3.4) | 0.7 **UL^7-90^**  (0.2) | 0.4 **UL^30^**  (0.1) | 0.6 **UL^30-90^**  (0.2) |
| **LIRI day 3** | 32 **US^3^L^7-90^**  (3.4) | 34  (4.2) | 1.2  (0.3) | 1.0  (0.5) | 0.6 **UL^30-90^**  (0.2) |
| **LIRI day 7** | 13 **L^30-90^**  (5.0) | 60 **UL^1-3,30^**  (8.7) | 2.3  (0.4) | 1.0  (0.4) | 0.8 **U**  (0.2) |
| **LIRI day 30** | ND | 35  (4.3) | 4.1  (1.2) | 2.3  (0.7) | 2.4  (0.8) |
| **LIRI day 90** | ND | 56 **US^90^L^1-3,30^**  (5.3) | 1.4  (0.3) | 0.6 **S^90^**  (0.1) | 1.3  (0.3) |

Neutrophils (N), macrophages (M), helper T-lymphocytes (CD5^+^CD4^+^), cytotoxic T-lymphocytes (CD5^+^CD8^+^), and B-lymphocytes (CD45RA^+^) in left BALf, presented as a percentage of total measured cells.

BALf=Broncho-Alveolar Lavage fluid; LIRI=Lung Ischemia-Reperfusion Injury; SEM=Standard-Error of the Mean; ND=Not Detectable

U=P<0.05 versus unoperated animals

S^x-y^=P<0.05 versus sham-operated animals from day x until day y

L^x-y^=P<0.05 versus LIRI animals from day x until day y

**Table 4B.** The total number of inflammatory cells (mean (SEM)) in left

| **Left BALf** | **Number of Cells (*1000)** | | | | |
| --- | --- | --- | --- | --- | --- |
|  | **N** | **M** | **CD4** | **CD8** | **CD45** |
| **Unoperated** | 24  (9.9) | 156  (32) | 9.2  (2.3) | 4.0  (1.0) | 10.0  (2.3) |
| **Sham day 1** | 164  (70) | 223  (44) | 7.6  (5.1) | 4.8  (3.6) | 8.4  (7.0) |
| **Sham day 3** | 70  (18) | 515 **S^90^**  (173) | 11.0 **S^90^**  (2.0) | 4.1  (1.6) | 2.5 **US^30^**  (0.5) |
| **Sham day 7** | 18  (4.6) | 246  (70) | 7.9  (2.6) | 3.2  (1.1) | 3.3 **US^30^**  (0.9) |
| **Sham day 30** | ND | 95  (34) | 11.0  (2.6) | 4.5  (2.2) | 11.7  (3.8) |
| **Sham day 90** | ND | 129  (25) | 4.9  (1.4) | 1.4 **U**  (0.3) | 7.1  (2.4) |
| **LIRI day 1** | 1,906 **US^1^L^7-90^**  (505) | 226  (58) | 14.0  (3.8) | 15.3 **UL^90^**  (3.6) | 11.9  (4.7) |
| **LIRI day 3** | 1,079 **US^3^L^7-90^**  (251) | 1,221 **S^3^L^1,30-90^**  (205) | 90.0 **US^3^L^1,30-90^**  (27.1). | 46.6 **US^3^L^30-90^**  (14.2) | 31.2 **US^3^L^30^**  (10.6) |
| **LIRI day 7** | 222  **S^7^L^30-90^**  (90) | 978 **US^7^L^1,30^**  (212) | 50.0 **US^7^L^1,90^**  (15.1) | 23.0  (8.7) | 20.0 **S^7^L^30^**  (5.3) |
| **LIRI day 30** | ND | 204  (50) | 23.4  (8.9) | 13.6  (5.4) | 7.5  (2.6) |
| **LIRI day 90** | ND | 497 **US^90^L^1,30^**  (96) | 11.3  (3.1) | 4.2 **S^90^**  (0.9) | 11.7  (3.3) |

broncho-alveolar lavage fluid.

Neutrophils (N), macrophages (M), helper T-lymphocytes (CD5^+^CD4^+^), cytotoxic T-lymphocytes (CD5^+^CD8^+^), and B-lymphocytes (CD45RA^+^) in left BALf, presented as total number of cells.

BALf=Broncho-Alveolar Lavage fluid; LIRI=Lung Ischemia-Reperfusion Injury; SEM=Standard-Error of the Mean; ND=Not Detectable

U=P<0.05 versus unoperated animals

S^x-y^=P<0.05 versus sham-operated animals from day x until day y

L^x-y^=P<0.05 versus LIRI animals from day x until day y

**Table 5A. The percentage of inflammatory cells (mean (SEM)) in right broncho-alveolar lavage fluid.**

| **Right BALf** | **% Cells** | | | | |
| --- | --- | --- | --- | --- | --- |
|  | **N** | **M** | **CD4** | **CD8** | **CD45** |
| **Unoperated** | 1.4  (0.7) | 58  (7.3) | 1.1  (0.3) | 0.6  (0.2) | 2.2  (0.6) |
| **Sham day 1** | 20 **U**  (8.0) | 53  (9.0) | 0.7  (0.2) | 0.3  (0.1) | 0.8  (0.3) |
| **Sham day 3** | 11 **U**  (4.1) | 68  (7.3) | 0.9  (0.5) | 0.6  (0.4) | 0.7  (0.2) |
| **Sham day 7** | 11 **U**  (4.6) | 74  (4.1) | 1.3  (0.3) | 0.4  (0.1) | 0.6  (0.2) |
| **LIRI day 1** | 25 **UL^7^**  (5.2) | 39  (4.6) | 0.5  (0.2) | 0.3  (0.1) | 0.4  (0.1) |
| **LIRI day 3** | 25 **US^3^L^7^**  (3.1) | 49 **S^3^**  5.2) | 1.0  (0.3) | 0.6  (0.3) | 0.8  (0.2) |
| **LIRI day 7** | 5.9  (2.3) | 67 **L^1-3^**  (5.1) | 1.1  (0.3) | 0.2  (0.1) | 0.8  (0.2) |

Neutrophils (N), macrophages (M), helper T-lymphocytes (CD5^+^CD4^+^), cytotoxic T-lymphocytes (CD5^+^CD8^+^), and B-lymphocytes (CD45RA^+^) in right BALf, presented as a percentage of total measured cells.

BALf=Broncho-Alveolar Lavage fluid; LIRI=Lung Ischemia-Reperfusion Injury; SEM=Standard-Error of the Mean

U=P<0.05 versus unoperated animals

S^x-y^=P<0.05 versus sham-operated animals from day x until day y

L^x-y^=P<0.05 versus LIRI animals from day x until day y

**Table 5B. The total number of inflammatory cells (mean (SEM)) in right broncho-alveolar lavage fluid.**

| **Right BALf** | **Number of Cells (*1,000)** | | | | |
| --- | --- | --- | --- | --- | --- |
|  | **N** | **M** | **CD4** | **CD8** | **CD45** |
| **Unoperated** | 13  (5) | 1,917  (384) | 8.1  (2.1) | 4.5  (1.3) | 12.5  (2.9) |
| **Sham day 1** | 90  (41) | 1,500  (188) | 7.5  (3.5) | 3.3  (1.6) | 11.3  (7.3) |
| **Sham day 3** | 40  (18) | 6,299  (1,510) | 4.3  (1.1) | 2.5  (1.4) | 9.2  (2.5) |
| **Sham day 7** | 28  (8) | 6,394  (1,011) | 4.7  (1.3) | 1.2  (0.4) | 4.0  (2.2) |
| **LIRI day 1** | 178 **U**  (41) | 1,663  (479) | 4.8  (1.0) | 3.6  (0.9) | 3.6  (0.6) |
| **LIRI day 3** | 428 **US^3^L^7^**  (156) | 4,612 **UL^1^**  (1,293) | 42.5 **US^3^L^1^**  (14.5) | 34.1 **US^3^L^1,7^**  (19.3) | 28.0  (11.7) |
| **LIRI day 7** | 96 **U**  (40) | 3,575 **US^7^L^1-3^**  (970) | 16.9 **S^7^**  (5.4) | 3.9  (1.6) | 12.5  (3.1) |

Neutrophils (N), macrophages (M), helper T-lymphocytes (CD5^+^CD4^+^), cytotoxic T-lymphocytes (CD5^+^CD8^+^), and B-lymphocytes (CD45RA^+^) in right BALf, presented as total number of cells.

BALf=Broncho-Alveolar Lavage fluid; LIRI=Lung Ischemia-Reperfusion Injury; SEM=Standard-Error of the Mean

U=P<0.05 versus unoperated animals

S^x-y^=P<0.05 versus sham-operated animals from day x until day y

L^x-y^=P<0.05 versus LIRI animals from day x until day y

**Table 6A. The percentage of inflammatory cells (mean (SEM)) in left lung tissue**

| **Left Lung tissue** | **% Cells** | | | | |
| --- | --- | --- | --- | --- | --- |
|  | **N** | **M** | **CD4** | **CD8** | **CD45** |
| **Unoperated** | 2.4  (0.8) | 10  (2.8) | 2.5  (0.4) | 1,9  (0.2) | 4.9  (0.4) |
| **Sham day 1** | 11 **US^3,30^**  (4.2) | 29 **US^30-90^**  (3.9) | 1.0 **US^3,30-90^**  (0.2) | 0.7 **US^30-90^**  (0.2) | 1.8 **US^3,30^**  (0.5) |
| **Sham day 3** | 3.0  (1.1) | 30 **US^30-90^**  (4.1) | 2.5  (0.4) | 1.6  (0.4) | 4.6  (1.2) |
| **Sham day 7** | 10  (6.0) | 41 **US^30-90^**  (6.9) | 1.7  (0.5) | 1.0 **US^30-90^**  (0.3) | 1.4 **US^3,30^**  (0.5) |
| **Sham day 30** | 1.7  (0.5) | 13  (3.6) | 3.2 **S^90^**  (0.3) | 2.2  (0.3) | 4.1  (0.5) |
| **Sham day 90** | 3.9  (1.2) | 13  (3.9) | 1.8  (0.2) | 1.8  (0.2) | 2.8 **US^30^**  (0.6) |
| **LIRI day 1** | 19 **UL^7-90^**  (4.9) | 29 **UL^30^**  (3.1) | 1.0 **UL^30-90^**  (0.1) | 1.2  (0.3) | 3.2  (0.6) |
| **LIRI day 3** | 18 **US^3^L^7-90^**  (4.4) | 37 **UL^30^**  (5.9) | 1.4 **U**  (0.3) | 0.9 **UL^30-90^**  (0.2) | 2.0 **US^3^**  (0.3) |
| **LIRI day 7** | 3.5  (0.6) | 36 **UL^30-90^**  (5.5) | 1.3 **UL^30-90^**  (0.2) | 0.6 **UL^30-90^**  (0.2) | 1.2 **UL^1-90^**  (0.2) |
| **LIRI day 30** | 1.5  (0.3) | 16  (1.6) | 2.1 **S^30^**  (0.3) | 1.6  (0.2) | 2.4 **US^30^**  (0.1) |
| **LIRI day 90** | 2.8  (0.8) | 20 **U**  (3.0) | 2.1  (0.2) | 1.7  (0.2) | 3.2  (0.6) |

Neutrophils (N), and macrophages (M), helper T-lymphocytes (CD5^+^CD4^+^), Cytotoxic T-lymphocytes (CD5^+^CD8^+^), and B-lymphocytes (CD45RA^+^) in left lung tissue, presented as a percentage of total measured cells.

LIRI=Lung Ischemia-Reperfusion Injury; SEM=Standard-Error of the Mean

U=P<0.05 versus unoperated animals

S^x-y^=P<0.05 versus sham-operated animals from day x until day y

L^x-y^=P<0.05 versus LIRI animals from day x until day y

**Table 6B. The total number of inflammatory cells (mean (SEM)) in left lung tissue**

| **Left Lung tissue** | **Number of Cells (*1,000)** | | | | |
| --- | --- | --- | --- | --- | --- |
|  | **N** | **M** | **CD4** | **CD8** | **CD45** |
| **Unoperated** | 596  (166) | 596  (166) | 136  (22) | 104  (14) | 263  (20) |
| **Sham day 1** | 774  (77) | 774  (77) | 57 **US^3,30^**  (17) | 32 **US^30-90^**  (9) | 95 **U**  (53) |
| **Sham day 3** | 3,796  (1,006) | 3,796 **US^1,30-90^**  (1,006) | 196  (56) | 107  (35) | 438 **S^1,90^**  (99) |
| **Sham day 7** | 4,531  (633) | 4,531 **US^1,30-90^**  (633) | 146  (40) | 147  (54) | 260  (118) |
| **Sham day 30** | 595  (198) | 595  (198) | 137  (15) | 92  (6) | 191  (33) |
| **Sham day 90** | 756  (299) | 756  (299) | 93  (20) | 93  (19) | 138 **U**  (31) |
| **LIRI day 1** | 3,353 **UL^7-90^**  (568) | 3,353 **US^1^L^30-90^**  (568) | 153 **S^1^L^3,90^**  (32) | 225 **US^1^L^30-90^**  (47) | 407 **S^1^L^7-90^**  (59) |
| **LIRI day 3** | 6,862 **US^3^L^7-90^**  (1,555) | 6,862 **UL^7-90^**  (1,555) | 445 **UL^7-90^**  (94) | 331 **US^3^ L^7-90^**  (54) | 430 **L^7-90^**  (66) |
| **LIRI day 7** | 2,649  (705) | 2,649 **US^7^L^30-90^**  (705) | 102 **L^90^**  (17) | 95  (39) | 147  (51) |
| **LIRI day 30** | 701  (73) | 701  (73) | 91 **S^30^L^90^**  (12) | 74 **L^90^**  (10) | 103 **US^30^**  (10) |
| **LIRI day 90** | 633  (148) | 633  (148) | 50 **U**  (8) | 41 **US^90^**  (7) | 93 **U**  (28) |

Neutrophils (N), and macrophages (M), helper T-lymphocytes (CD5^+^CD4^+^), Cytotoxic T-lymphocytes (CD5^+^CD8^+^), and B-lymphocytes (CD45RA^+^) in left lung tissue, presented as total number of cells.

LIRI=Lung Ischemia-Reperfusion Injury; SEM=Standard-Error of the Mean

U=P<0.05 versus unoperated animals

S^x-y^=P<0.05 versus sham-operated animals from day x until day y

L^x-y^=P<0.05 versus LIRI animals from day x until day y

**Table 7A. The percentage of inflammatory cells (mean (SEM)) in right lung tissue**

| **Right Lung tissue** | **% Cells** | | | | |
| --- | --- | --- | --- | --- | --- |
|  | **N** | **M** | **CD4** | **CD8** | **CD45** |
| **Unoperated** | 2.2  (0.4) | 19  (1.9) | 3.5  (0.7) | 2.4  (0.4) | 4.7  (1.1) |
| **Sham day 1** | 6.1 **U**  (0.8) | 31  (3.8) | 0.8 **U**  (0.1) | 0.5 **U**  (0.1) | 2.9  (0.6) |
| **Sham day 3** | 5.5 **U**  (1.6) | 29  (5.7) | 1.8  (0.5) | 1.1 **U**  (0.4) | 3.6  (0.7) |
| **Sham day 7** | 9.1  (4.5) | 36  (4.2) | 2.0  (0.5) | 1.0  (0.3) | 1.7  (0.6) |
| **LIRI day 1** | 8.4 **U**  (1.7) | 29  (2.9) | 0.7 **UL^3-7^**  (0.1) | 0.8 **U**  (0.2) | 1.8  (0.4) |
| **LIRI day 3** | 9.5 **U**  (4.8) | 31  (4.6) | 1.3 **U**  (0.2) | 0.8 **U**  (0.2) | 2.4  (0.5) |
| **LIRI day 7** | 3.7  (1.3) | 32  (5.2) | 1.8 **U**  (0.3) | 0.9 **U** (0.2) | 1.9  (0.4) |

Neutrophils (N), and macrophages (M), helper T-lymphocytes (CD5^+^CD4^+^), Cytotoxic T-lymphocytes (CD5^+^CD8^+^), and B-lymphocytes (CD45RA^+^) in right lung tissue, presented as a percentage of total measured cells.

LIRI=Lung Ischemia-Reperfusion Injury; SEM=Standard-Error of the Mean

U=P<0.05 versus unoperated animals

S^x-y^=P<0.05 versus sham-operated animals from day x until day y

L^x-y^=P<0.05 versus LIRI animals from day x until day y

**Table 7B. The total number of inflammatory cells (mean (SEM)) in right lung tissue**

| **Right Lung tissue** | **Number of Cells (*1000)** | | | | |
| --- | --- | --- | --- | --- | --- |
|  | **N** | **M** | **CD4** | **CD8** | **CD45** |
| **Unoperated** | 280  (76) | 1,917  (384) | 314  (84) | 232  (49) | 508  (144) |
| **Sham day 1** | 334  (43) | 1,500  (188) | 43 **US^3-7^**  (10) | 27 **US^3-7^**  (6) | 152 **US^3^**  (37) |
| **Sham day 3** | 684 **U**  (101) | 6,299 **US^1^**  (1,510) | 413  (104) | 271  (74) | 691  (180) |
| **Sham day 7** | 340  (106) | 6,394 **US^1^**  (1,011) | 271  (38) | 156  (40) | 249  (83) |
| **LIRI day 1** | 993 **US^1^**  (256) | 2,663 **S^1^**  (479) | 89 **US^1^L^3-7^**  (15) | 104 **US^1^**  (15) | 224  (47) |
| **LIRI day 3** | 735  (147) | 4,612  (1,293) | 249  (53) | 183  (46) | 442  (103) |
| **LIRI day 7** | 581  (153) | 3,575  (970) | 422  (127) | 233  (77) | 314  (95) |

Neutrophils (N), and macrophages (M), helper T-lymphocytes (CD5^+^CD4^+^), Cytotoxic T-lymphocytes (CD5^+^CD8^+^), and B-lymphocytes (CD45RA^+^) in right lung tissue, presented as total number of cells.

LIRI=Lung Ischemia-Reperfusion Injury; SEM=Standard-Error of the Mean

U=P<0.05 versus unoperated animals

S^x-y^=P<0.05 versus sham-operated animals from day x until day y

L^x-y^=P<0.05 versus LIRI animals from day x until day y

**Table 8A. The percentage of inflammatory cells (mean (SEM)) in thoracic lymph nodes**

| **TLN** | **% Cells** | | |
| --- | --- | --- | --- |
|  | **CD4** | **CD8** | **CD45** |
| **Unoperated** | 21  (2.2) | 11  (1.2) | 28  (2.2) |
| **Sham day 1** | 27 **S^3,90^**  (1.6) | 10  (0.9) | 31  (2.3) |
| **Sham day 3** | 19  (2.4) | 11  (0.9) | 33 **S^90^**  (3.7) |
| **Sham day 7** | 20  (2.9) | 12  (2.0) | 26  (2.8) |
| **Sham day 30** | 27 **S^3-7,90^**  (1.1) | 17 **US^1,3^**  (1.0) | 26  (3.1) |
| **Sham day 90** | 21  (1.7) | 15 **S^1^**  (1.9) | 26  (1.8) |
| **LIRI day 1** | 24  (1.5) | 13  (1.1) | 31  (2.1) |
| **LIRI day 3** | 19  (1.7) | 13  (1.1) | 40 **UL^1,7-90^**  (2.6) |
| **LIRI day 7** | 19  (1.8) | 10  (1.3) | 34 **S^7^L^30-90^**  (1.9) |
| **LIRI day 30** | 25  (2.6) | 14  (1.4) | 27  (1.9) |
| **LIRI day 90** | 28 **US^90^L^3-7^**  (1.9) | 14  (0.7) | 25  (1.9) |

Helper T-lymphocytes (CD5^+^CD4^+^), Cytotoxic T-lymphocytes (CD5^+^CD8^+^), and B-lymphocytes (CD45RA^+^) in TLN, presented as a percentage of total measured cells.

LIRI=Lung Ischemia-Reperfusion Injury; SEM=Standard-Error of the Mean; TLN=Thoracic lymph nodes

U=P<0.05 versus unoperated animals

S^x-y^=P<0.05 versus sham-operated animals from day x until day y

L^x-y^=P<0.05 versus LIRI animals from day x until day y

**Table 8B. The total number of inflammatory cells (mean (SEM)) in thoracic lymph nodes**

| **TLN** | **Number of Cells (*1000)** | | |
| --- | --- | --- | --- |
|  | **CD4** | **CD8** | **CD45** |
| **Unoperated** | 1,886  (248) | 1,087  (200) | 2,391  (323) |
| **Sham day 1** | 2,444  (696) | 891  (204) | 2,430  (577) |
| **Sham day 3** | 3,093  (637) | 1,743 **S^1,90^**  (301) | 5,783 **US^1,30-90^**  (925) |
| **Sham day 7** | 2,825  (938) | 2,221  (597) | 3,304  (831) |
| **Sham day 30** | 2,944  (617) | 1,838  (416) | 1,653  (444) |
| **Sham day 90** | 1,215 **S^3,30^**  (308) | 776  (272) | 2,094  (738) |
| **LIRI day 1** | 2,784  (461) | 1,440  (244) | 3,826  (1,057) |
| **LIRI day 3** | 5,670 **UL^1,90^**  (1,025) | 4,281 **US^3^L^1,30,90^**  (975) | 11,868 **UL^1,30-90^**  (2,950) |
| **LIRI day 7** | 5,130 **U**  (1,085) | 3,055 **U**  (798) | 12,793 **US^7^L^1,30-90^**  (2,883) |
| **LIRI day 30** | 3,860 **U**  (824) | 1,954 **U**  (329) | 3,930 **S^30^**  (910) |
| **LIRI day 90** | 2,934 **S^90^**  (488) | 1,747 **S^90^**  (272) | 2,956  (618) |

Helper T-lymphocytes (CD5^+^CD4^+^), Cytotoxic T-lymphocytes (CD5^+^CD8^+^), and B-lymphocytes (CD45RA^+^), presented as total number of cells in TLN.

LIRI=Lung Ischemia-Reperfusion Injury; SEM=Standard-Error of the Mean; TLN=Thoracic lymph nodes

U=P<0.05 versus unoperated animals

S^x-y^=P<0.05 versus sham-operated animals from day x until day y

L^x-y^=P<0.05 versus LIRI animals from day x until day y

Table 9. Histologic score of the left lung

|  | | **IAE** | | | | | **SE** | | | | | **IAH** | | | | **FIB** | | | | **ATL** | | | | **OC** | | | |
| --- | --- | --- | --- | --- | --- | --- | --- | --- | --- | --- | --- | --- | --- | --- | --- | --- | --- | --- | --- | --- | --- | --- | --- | --- | --- | --- | --- |
|  |  | **A** | **B** | **C** | **D** | | **A** | | **B** | **C** | **D** | **A** | **B** | **C** | **D** | **A** | **B** | **C** | **D** | **A** | **B** | **C** | **D** | **N** | **E** | **F** | **R** |
| **Unoperated** | | 3 |  |  |  | | 3 | |  |  |  | 3 |  |  |  | 3 |  |  |  | 3 |  |  |  | 3 |  |  |  |
| **Sham day 1** | | 3 |  |  |  | | 3 | |  |  |  | 3 |  |  |  | 3 |  |  |  | 2 | 1 |  |  | 3 |  |  |  |
| **Sham day 3** | | 1 | 1 | 1 |  | | 2 | | 1 |  |  | 3 |  |  |  | 3 |  |  |  | 3 |  |  |  | 1 | 1 |  | 1 |
| **Sham day 7** | | 3 |  |  |  | | 3 | |  |  |  | 3 |  |  |  | 3 |  |  |  | 3 |  |  |  | 3 |  |  |  |
| **Sham day 30** | | 3 |  |  |  | | 2 | | 1 |  |  | 3 |  |  |  | 3 |  |  |  | 3 |  |  |  | 2 |  |  | 1 |
| **Sham day 90** | | 3 |  |  |  | | 3 | |  |  |  | 3 |  |  |  | 3 |  |  |  | 3 |  |  |  | 3 |  |  |  |
| **LIRI day 1** | |  |  |  | 3 | |  | | 3 |  |  |  | 3 |  |  | 3 |  |  |  | 3 |  |  |  |  | 3 |  |  |
| **LIRI day 3** | |  | 2 |  | 1 | |  | | 1 | 2 |  | 1 | 1 | 1 |  | 1 | 2 |  |  |  | 3 |  |  |  | 3 | 2 |  |
| **LIRI day 7** | | 3 |  |  |  | | 3 | |  |  |  | 3 |  |  |  | 1 | 1 | 1 |  |  |  | 2 | 1 |  |  | 3 |  |
| **LIRI day 30** | | 3 |  |  |  | | 3 | |  |  |  | 3 |  |  |  |  | 3 |  |  |  |  | 3 |  |  |  | 3 |  |
| **LIRI day 90** | | 3 |  |  |  | | 2 | | 1 |  |  | 3 |  |  |  | 1 | 2 |  |  | 1 |  | 2 |  | 1 |  | 2 |  |
| **Scoring** | | | | | | **Classification** | | | | | | | | | |  | | | | | | | | | | | |
| **A** | None | | | | | **N** | | Normal | | | | | | | |  | | | | | | | | | | | |
| **B** | Mild/scattered | | | | | **E** | | Exsudative | | | | | | | |  | | | | | | | | | | | |
| **C** | Moderate/occasional | | | | | **F** | | Fibroproliferative | | | | | | | |  | | | | | | | | | | | |
| **D** | Severe/frequent | | | | | **R** | | Resolving | | | | | | | |  | | | | | | | | | | | |

HE sections of 3 animals per group were scored for intra-alveolar edema (IAE), septal edema (SE), intra-alveolar hemorrhage (IAH), fibrosis (FIB), atelectasis (ATL), and overall classification (OC). Represented are the number of animals within each group with their respective parameter score Lungs were classified as N) normal if no abnormalities were seen, E) exsudative if pulmonary edema and/or hyaline membranes were present, F) fibroproliferative, if activated fibroblasts and/or proliferating alveolar type II cells were found, and R) resolving if injury was on return to normal. LIRI: Lung ischemia-reperfusion injury.

**Table 10.** Histologic score of the left lung

| **Histology** | | **INF** | | | | | | **INF CLS** | | | |
| --- | --- | --- | --- | --- | --- | --- | --- | --- | --- | --- | --- |
|  |  | **A** | **B** | **C** | | | **D** | **L** | **H** | **G** | **M** |
| **Unoperated** | |  | 3 |  | | |  | 3 |  |  |  |
| **Sham day 1** | | 2 | 1 |  | | |  | 1 |  |  |  |
| **Sham day 3** | |  | 3 |  | | |  | 1 | 2 |  |  |
| **Sham day 7** | |  | 3 |  | | |  | 1 | 2 |  |  |
| **Sham day 30** | | 2 | 1 |  | | |  | 1 |  |  |  |
| **Sham day 90** | | 1 | 2 |  | | |  | 2 |  |  |  |
| **LIRI day 1** | |  | 2 | 1 | | |  |  |  | 1 | 2 |
| **LIRI day 3** | |  |  | 2 | | | 1 |  | 3 | 1 |  |
| **LIRI day 7** | |  |  | 2 | | | 1 | 2 | 3 |  |  |
| **LIRI day 30** | |  |  | 3 | | |  |  | 3 |  |  |
| **LIRI day 90** | |  | 1 | 2 | | |  | 1 | 2 |  |  |
| **Scoring** | | | | | **Inflammation** | | | | | | |
| **A** | None | | | | **L** | Lymhocytic | | | | | |
| **B** | Mild/scattered | | | | **H** | Histiocytic | | | | | |
| **C** | Moderate/occasional | | | | **G** | Granulocytic | | | | | |
| **D** | Severe/frequent | | | | **M** | Mixed | | | | | |

HE sections of 3 animals per group were scored for inflammation severity (INF), and type of inflammatory cells (INF CLS). Represented are the number of animals within each group with the respective parameter score. Some groups may contain more than 3 scores, since some animals had mixed inflammatory patterns. LIRI: Lung ischemia-reperfusion injury.
